# *Giardia*-specific cellular immune responses in post-giardiasis chronic fatigue syndrome

**DOI:** 10.1186/s12865-017-0190-3

**Published:** 2017-01-28

**Authors:** Kurt Hanevik, Einar Kristoffersen, Kristine Mørch, Kristin Paulsen Rye, Steinar Sørnes, Staffan Svärd, Øystein Bruserud, Nina Langeland

**Affiliations:** 1 Department of Clinical Science, Lab-building 8.floor, University of Bergen, N-5021 Bergen, Norway; 2 Center for Tropical Infectious Diseases, Haukeland University Hospital, Bergen, Norway; 3 Department of immunology and transfusion medicine, Haukeland University Hospital, Bergen, Norway; 4 Department of Cell and Molecular biology, Uppsala University, Uppsala, Sweden

**Keywords:** *Giardia*, T cell, Chronic fatigue syndrome, Antigen-specific, Immune response, sCD40L

## Abstract

**Background:**

The role of pathogen specific cellular immune responses against the eliciting pathogen in development of post-infectious chronic fatigue syndrome (PI-CFS) is not known and such studies are difficult to perform. The aim of this study was to evaluate specific anti-*Giardia* cellular immunity in cases that developed CFS after *Giardia* infection compared to cases that recovered well. Patients reporting chronic fatigue in a questionnaire study three years after a *Giardia* outbreak were clinically evaluated five years after the outbreak and grouped according to Fukuda criteria for CFS and idiopathic chronic fatigue. *Giardia* specific immune responses were evaluated in 39 of these patients by proliferation assay, T cell activation and cytokine release analysis. 20 *Giardia* exposed non-fatigued individuals and 10 healthy unexposed individuals were recruited as controls.

**Results:**

Patients were clinically classified into CFS (*n* = 15), idiopathic chronic fatigue (*n* = 5), fatigue from other causes (*n* = 9) and recovered from fatigue (*n* = 10). There were statistically significant antigen specific differences between these *Giardia* exposed groups and unexposed controls. However, we did not find differences between the *Giardia* exposed fatigue classification groups with regard to CD4 T cell activation, proliferation or cytokine levels in 6 days cultured PBMCs. Interestingly, sCD40L was increased in patients with PI-CFS and other persons with fatigue after *Giardia* infection compared to the non-fatigued group, and correlated well with fatigue levels at the time of sampling.

**Conclusion:**

Our data show antigen specific cellular immune responses in the groups previously exposed to *Giardia* and increased sCD40L in fatigued patients.

**Electronic supplementary material:**

The online version of this article (doi:10.1186/s12865-017-0190-3) contains supplementary material, which is available to authorized users.

## Background

The causes and underlying mechanisms for development of chronic fatigue syndrome (CFS) remain unresolved. In some cases the condition is elicited by an infection, with mononucleosis due to Epstein Barr infection (EBV) being the most well-known [1]. However, it is also described to occur following a number of other infections [1]. When the onset of CFS is associated with an acute infection, it can be termed post-infectious (PI) and many researchers have focused on measures of the immune system in the quest to understand mechanisms and identify biomarkers [2–6].

It is likely that the nature of the host reaction to the specific eliciting pathogens is implicated in development of PI-CFS. This is inherently difficult to study, as patients often present at a late stage where it is difficult to ascertain the specific eliciting pathogen and it is challenging to gather enough patients of a specific etiology to perform such studies.

An opportunity to investigate potential differences in the magnitude or quality of host responses towards the eliciting pathogen arose when a fraction of *Giardia* assemblage B infected persons developed post-infectious chronic fatigue (CF) after a large waterborne outbreak in Bergen, Norway in 2004 [7]. Most of these individuals also had co-morbid functional gastrointestinal disorders (FGID) elicited by the *Giardia* infection. Post-giardiasis chronic fatigue had not previously been described in the literature. It was the clinical follow-up of referred patients with persisting symptoms after successful *Giardia* treatment that informed the choice of questionnaires which were sent to all laboratory-confirmed cases two years after the outbreak [8], and at three years when we also included a control group [9]. Severity of the primary *Giardia* illness was a risk factor for developing both FGID and CF [10]. It became clear that chronic fatigue symptoms were four times more prevalent in the *Giardia* exposed group compared to controls [9]. Five years after the outbreak, PI-CFS was found in 42% (22/53) among patients who had reported chronic fatigue in the questionnaire three year after the outbreak [11].

In the present study we aimed to evaluate if *Giardia* specific immune responses such as proliferative capacity, CD4 T cell activation and cytokine profiles, were associated with development of CFS in this group of carefully clinically characterized persons. Responses were compared with a *Giardia* exposed group without fatigue, and with *Giardia*-naïve controls, in order to allow interpretation of the data with respect to *Giardia* exposure and specificity of the responses.

## Methods

### Study populations

**Fig. 1:**
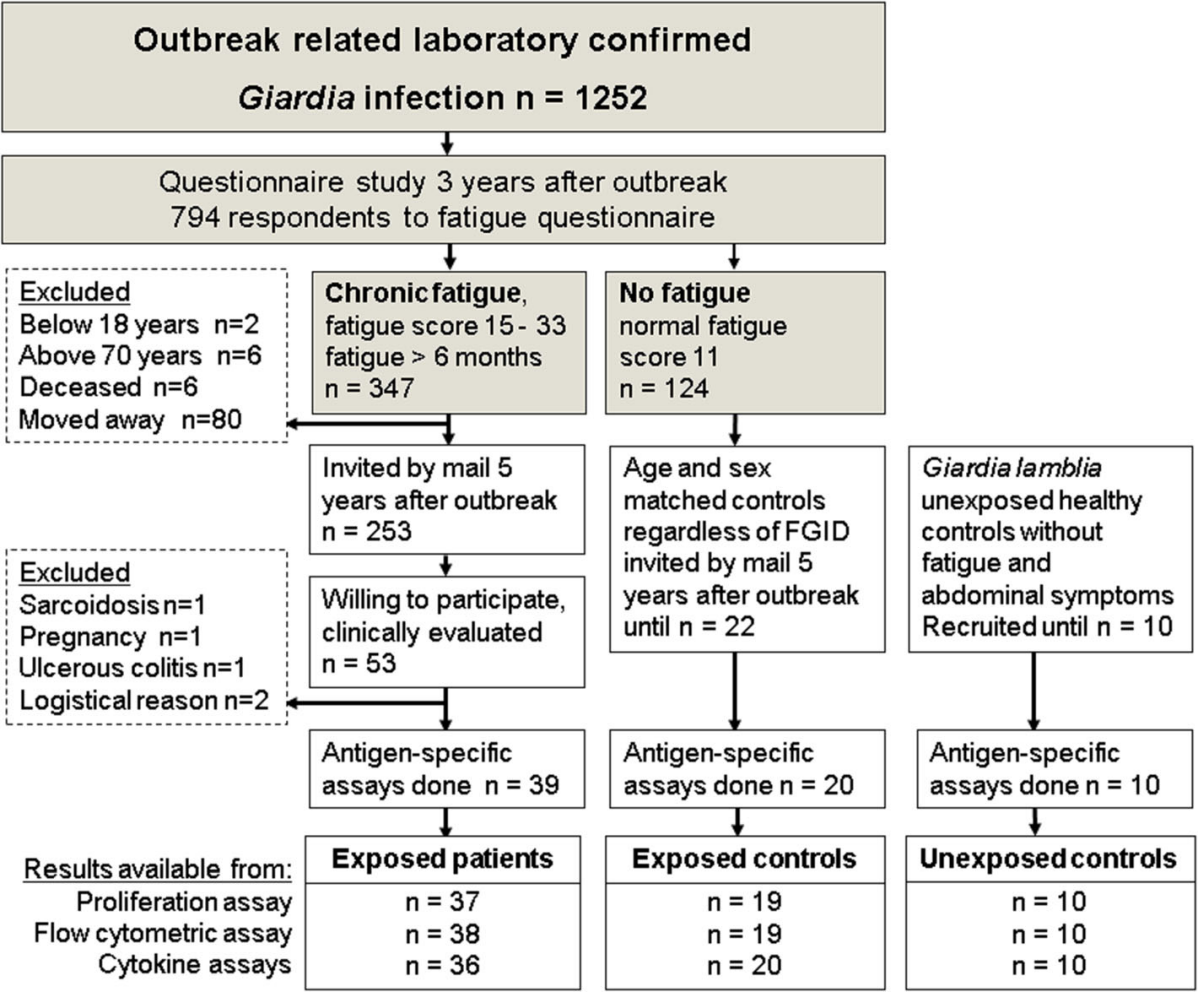
Selection of the patient poulation for the present study

Participants were recruited based on responses to a questionnaire mailed three years (in 2007) after the outbreak to all persons with laboratory confirmed giardiasis during the outbreak [9]. Patients who reported chronic fatigue in this questionnaire were invited to participate in a thorough clinical evaluation and screening two years later (in 2009). Fifty-three individuals agreed to participate, and went through a clinical evaluation by specialists in internal medicine, psychiatry and neurology. They were evaluated for CFS or idiopathic chronic fatigue (ICF) according to the 1994 Fukuda criteria [12]. Those fulfilling the criteria, and had an onset of symptoms related to the *Giardia* infection, were categorized as PI-CSF or PI-ICF. Patients with sleep apnea syndrome, significant depression or anxiety disorders that could plausibly explain their fatigue were termed “fatigue other cause”. Individuals who had recovered well from the fatigue condition they had reported in the questionnaire two years previously were termed “fatigue recovered”. Five patients were excluded from this study after clinical evaluation (Fig. 1).

Two control groups were recruited; 22 individuals with normal fatigue score (=11) in the 2007 questionnaire (exposed, no-PI-fatigue group), and 10 healthy individuals not affected by the outbreak and without particular fatigue or abdominal symptoms (unexposed healthy controls) (Fig. 1). All participants were HIV negative and were not taking immunomodulatory medications or antibiotics.

### Sampling and questionnaires

Participants were screened with a battery of routine blood tests and a magnetic resonance imaging (MRI) brain scan. Blood samples were taken between 08 am and 09 am after overnight fast and analyzed in parallel during the same period. Immunophenotyping results from these investigations have been reported previously [13]. Fecal samples were obtained and screened with microscopic examination and *Giardia* 18S PCR [14] of feces to rule out chronic giardiasis.

A total of 69 of the study participants underwent clinical characterization and were subject to one or more of the *Giardia*-specific immune response analyses presented in this paper. Some assays could not be done in all patients due to limited cell numbers (Fig. 1). Sampling and assays were stratified across groups and samples were blinded to laboratory personnel to avoid analytical bias. The severity of fatigue at the time of sampling was evaluated in all participants by the Fatigue Questionnaire [15], a validated set of 11 questions addressing different aspects of fatigue. Comorbid abdominal symptoms were recorded by the commonly used Rome II questionnaire [16].

### Antigens

*The Giardia* antigens used in this study were made from culturing *Giardia* assemblage A strain WB-C6 (ATCC 50803) and assemblage B strain GS/M (ATCC 50581) in Diamond’s TYI-S-33 medium supplemented with bile as described previously[17]. Trophozoites were washed in PBS, treeze-thawed, then sonicated for 1 min at 20 W. After sentrifugation at 13000 x g, the supernatant containing soluble *Giardia* proteins was removed, and protein content was measured by the BCA protein assay kit (Pierce). Pilot testing showed that 10ug/mL of this mixed soluble *Giardia* antigens resulted in robust T cell responses and little background stimulation.

For the proliferation assay also a sterile filtered *Candida albicans* protein extract (403 skin prick test [Allergopharma]; 10 000 BU/mL), were added as an exploratory antigen and anti-CD3 + anti-CD28 as a control of T cell activation. Positive antigen controls were tuberculin purified protein derivate (PPD) (Statens Serum Institut) and *Salmonella typhi* LPS in all assays. Several concentrations were tested in pilot studies of all stimulation antigens and positive controls to optimize the assay.

### PBMC acquisition and antigen-specifc immunity assays

Peripheral blood mononuclear cells (PBMC) were isolated from BD Vacutainer Na-citrate CPT tubes (BD, Franklin Lakes, NJ, USA) by density gradient separation. After harvesting, the PBMC were washed twice in PBS and were cultured in the presence or absence of investigational or control antigens at 37 °C in a humidified atmosphere of 5% CO_2_for six days in X-vivo 15 serum-free culture medium supplemented with l-glutamin, gentamicin, and phenol red (BioWhittaker).Proliferative responses were measured in triplicates of stimulated cultured cells by adding ^3^H-thymidine (Amersham International) after five days, and harvesting 18 h later. IIncorporated radioactivity was analysed by liquid scintillation counting in a β-counter. Proliferation in stimulated and unstimulated cultures was determined as median counts per minute (cpm) of each triplicate.

Activation of T cells was evaluated after six days in culture. 100 μL supernatants were carefully harvested from wells of unstimulated and stimulated PBMCs and frozen at −80 °C for later cytokine analysis. The cultured cells were then analysed by flow cytometric measurement of activated T cell subsets as published previously [17]. Briefly, after washing with PBS, the cultured cell suspensions (50 μL) were stained for 30 min in the dark with combination of 5 fluorescent dyes: CD3-ECD (Beckman Coulter), CD8a-FITC and CD4-PerCP/Cy5.5 and the activation markers CD26-PE and CD25-PE/Cy7 (BioLegend). After staining, cells were washed once, resuspended in PBS-paraformaldehyde solution (1%) and analyzed the same day using a Beckman Coulter Cytomics FC 500 MPL flow cytometer. In a typical acquisition 7×10^4^ lymphocytes (min 2.3 ×10^4^, max 1.7 ×10^5^) were collected. The collected data were analyzed with FlowJo 7.6 software (Tree Star Inc, Ashland, OR, USA). Background responses in unstimulated cultures were adjusted for by subtracting these from responses in stimulated cultures.

Supernatants were kept frozen until analysis in three Bio-Plex assays (Bio-Rad Laboratories Inc., Hercules, CA, USA) for IFN-γ, TNF-α, IL-1β, IL-2, IL-4, IL-6, IL-9, IL-10, IL-13, IL-17A, IL-22, soluble (s) sCD40L, macrophage inflammatory protein 1 alpha (MIP-1α), MIP-1β, TGFβ1, TGFβ2, TGFβ3 and granulocyte macrophage colony-stimulation factor (GM-CSF) according to the manufacturer’s instructions. The cytokines TGFβ1, TGFβ2, TGFβ3 were analyzed in unstimulated and *Giardia* antigen stimulated cultures only. Observed concentrations (pg/ml) within the standard range were used for analysis. Values above the limit of quantitation were set to the highest value in the standard range, and values below this range were set to zero. For analytes where less than 50% of values were within range, further analyses were not done.

For both flow cytometric and cytokine assays, background responses in unstimulated cultures were subtracted from those in stimulated cultures for each participant. In some cases this gave negative values in the cytokine assay which were kept in the statistical analyses and interpreted as stimulant-induced decrease in production or increase in consumption of the relevant analyte.

### Statistical analysis

Unless otherwise stated the data are presented as median (standard deviation (SD)). Chi-squared tests were used for categorical comparisons between groups. Linear regression analysis was used for correlation between response parameters as well as fatigue scores and sCD40L data. Comparison of T cell activation, proliferation and cytokine data between exposed and unexposed groups was done using the Mann Whitney *U* test. The significance level was set at *p* <0.05. To reduce false positive findings due to multiple comparisons within the exposed groups, we used the Kruskal-Wallis test across all exposed groups first, and further testing between the no PI-fatigue and PI-CFS/PI-ICF groups with Mann Whitney *U* test was only performed for variables with a p value less than 0.05. IBM SPSS Statistics version 23 (IBM Corp, Armonk, USA) was used for statistical analysis.

## Results

### Patient characteristics

Patients and controls were grouped according to their clinical condition at the time of sampling. Data from 39 patients (median age 40.9 years (11.2) range 19–62, females 74%), 20 exposed controls (median age 39.5 (9.3) range 27–66, females 75%) and 10 unexposed controls (median age 43.3 (14.3) range 22–63, females 70%) were analyzed in this study. There were no significant differences in age (*p* = 0.06) or sex distribution (*p* = 0.61). Fatigued patients were categorized according to their clinical categories for analyses of antigen specific immune responses (Table 1). ICF and CFS were combined in the further analyses as they were seen as a common phenotype with difference only in severity of symptoms. The fatigue scores generally reflected the categorization obtained by careful clinical evaluation with the cases of CSF scoring higher than the ICF and fatigue by other cause groups. The patients who had recovered well still had a modestly elevated fatigue score compared to recruited exposed and unexposed controls.

**Table 1:** Characteristics of participant groups

	Age, median (SD)	Females, *n* (%)	Fatigue score in 2009, median (SD)	Total participants, *n*
*Giardia* exposed				
No PI-fatigue controls	39.5 (9.3)	15 (75)	11 (1.3)	20
PI-CFS	47.0 (8.6)	11 (73)	22.0 (5.3)	15
PI-ICF	35.0 (8.9)	4 (80)	20.0 (3.1)	5
Fatigue other cause	51.0 (15.6)	9 (100)	18.0 (5.1)	9
Recovered from fatigue	31.0 (5.8)	5 (50)	17.0 (3.3)	10
*Giardia* unexposed				
Healthy controls	39 (11.1)	7 (70)	11 (2.1)	10

*Abbreviations*: *PI* post-infectious, *CFS* chronic fatigue syndrome, *ICF* idiopathic chronic fatigue

#### *Giardia*-induced proliferation of immunocompetent cells

PMBC proliferated well in response to all stimuli included in this assay. A marked memory response was seen in the *Giardia* exposed groups that showed significantly stronger proliferation in response to the *Giardia* lysates compared to the unexposed group (Table 2). However, the proliferative responses to *Giardia* antigens did not differ significantly between the fatigue categories within the exposed group (Table 2). For the other specific antigen controls we found neither a difference with regard to previous exposure to *Giardia*, nor according to fatigue sequels. There was a trend towards decreased proliferation induced by the strong T cells activator anti-CD3antiCD28 among the fatigued groups compared to the no fatigued controls, but this did not attain significance (Table 2).

**Table 2:** Immune responses against *Giardia* and control antigens as measured by ^3^H-thymidine proliferation assay

	Exposed				Unexposed	*p*-values	
Stimulation agent	No PI-fatigue controls (*n* = 19)	PI-CFS & ICF (*n* = 19)	Fatigue other cause (*n* = 9)	Recovered from fatigue (*n* = 9)	Healthy controls (*n* = 10)	Exposed *n* = 56)vs unexposed (*n* = 10)	No PI-fatigue controls (*n*= 19) vs PI-CFS/ICF(*n* = 19)
Giardia ass A 10 μg/ml	12.8 (13.0)	15.3 (19.9)	20.3 (10.9)	15.1 (65.0)	3.5 (6.2)	0.001	ns*
Giardia ass B 10 μg/ml	12.2 (13.9)	13.3 (15.3)	23.3 (13.4)	15.3 (39.0)	3.5 (4.7)	<0.001	ns*
Tuberculin (PPD)10 μg/ml	26.3 (34.1)	34.0 (47.5)	54.9 (84.5)	42.5 (23.2)	27.7 (27.0)	ns	ns*
LP *S Stypi* 1 μg/ml	21.5 (18.6)	14.4 (13.2)	38.2 (34.3)	21.9 (19.5)	17.7 (10.5)	ns	ns*
C *albicans* 10 μg/ml	7.0 (9.1)	5.8 (11.9)	10.0 (32.0)	14.6 (6.0)	11.0 (9.4)	ns	ns*
aCD3aCD28(pos ctr)	70.6 (35.4)	51.4 (33.0)	51.8 (44.1)	51.8 (63.9)	60.1 (39.8)	ns	ns*

*KruskalWallis test across all exposed groups was not significant (below *p* = 0.05). ns = not significant

Proliferation responses are expressed as median stimulation indices (counts per minute(cpm) in stimulated triplicate cultures divided by cpm in unstimulated triplicate cultures) followed by standard deviation

#### *Giardia*-induced T cell expression of activation markers

Levels of activated CD4 T cells expressing CD25 and CD26 were significantly elevated in *Giardia* exposed groups compared to unexposed patients when stimulated with both *Giardia* assemblage A and assemblage B lysates (Table 3). The PPD and LPS control antigens induced similar levels of activated CD4 T cells in both exposed and unexposed groups. None of these stimuli induced differences between the exposed participants with or without fatigue sequels. The flow cytometric responses correlated well with proliferation responses to the same antigens with *p* <0.001 for assemblage A stimulation and *p* = 0.001 for assemblage B stimulated cultures.

**Table 3:** Immune responses against *Giardia* and control antigens as measured by the percentage of CD25CD26 positive CD4 T cells by flow-cytometry

	Exposed				Unexposed	*p*-values	
Stimulation agent	No PI-fatigue controls (*n* = 19)	PI-CFS & ICF (*n* = 18)	Fatigue other cause (*n* = 9)	Recovered from fatigue (*n* = 10)	Healthy controls (*n* = 10)	Exposed (*n* = 56)vs unexposed (*n* = 10)	No PI-fatigue controls (*n* = 19) vs PI-CFS/ICF(*n* = 19)
Giardia ass A 10 μg/ml	8.6 (5.8)	10.3 (7.7)	8.8 (5.0)	12.4 (8.1)	1.8 (3.9)	0.001	ns*
Giardia ass B 10 μg/ml	3.1 (2.2)	4.3 (7.3)	3.7 (3.9)	7.9 (5.7)	1.9 (1.6)	0.009	ns*
Tuberculin (PPD)10 μg/ml	6.6 (5.4)	11.3 (11.1)	9.2 (11.4)	13.6 (5.2)	10.0 (13.6)	ns	ns*
LPS *S. typi* 1 μg/ml	5.2 (5.5)	5.2 (9.6)	4.5 (2.5)	5.1 (2.9)	3.0 (14.6)	ns	ns*

The values are percentages of activated CD4 T cells out of all CD4 T cells in stimulated cultures subtracted by the percentage of activated CD4 T cells in unstimulated culture

*KruskalWallis test across all exposed groups was not significant (below *p* = 0.05). ns = not significant

#### *Giardia*-induced release of soluble mediators

In Table 4 the results of cytokines and sCD40L are given for levels of an array of cytokines and sCD40L in supernatants of PBMC stimulated for six days with *Giardia* assemblage B lysate. There were significant differences for most of these, except IL-4, IL-2, TGFβ2 and TGFβ3 between the *Giardia* exposed and unexposed participants. The same pattern was seen, but with weaker differences in responses to *Giardia* assemblage A. For PPD there were smaller, but significant differences only for IFNγ and sCD40L between exposed and unexposed groups, while for LPS stimulated supernatants no difference was found (Additional file 1: Table S1).

**Table 4:** Analyses of cytokines and sCD40L in supernatants of PBMC cultured for six days after stimulation with *Giardia* assemblage B lysate in persons exposed to this pathogen in the Bergen 2004 outbreak with or without fatigue sequels, and in unexposed controls

	Exposed				Unexposed	p-values	
Analyte	No PI-fatigue controls (*n* = 20)	PI-CFS & ICF (*n* = 19)	Fatigue other cause (*n* = 8)	Fully recovered (*n* = 9)	Healthy controls (*n* = 10)	Exposed (*n* = 56)vs unexposed (*n* = 10)	No PI-fatigue controls (*n* = 20) vs PI-CFS/ICF (*n* = 19)
IL-1b	8.4 (63.5)	23.7 (211)	10.0 (37.0)	37.3 (207)	0.8 (8.8)	<0.001	ns*
IL-4	4.1 (6.6)	4.9 (4.8)	-0.7 (7.4)	7.7 (5.6)	4.0 (29.4)	0.838	ns*
IL-6	496 (8364)	781 (18566)	957 (659)	741 (991)	85.3 (310)	<0.001	ns*
IL-10	19.7 (23.3)	25.2 (354)	22.7 (13.2)	25.1 (5.9)	7.1 (25.3)	<0.001	ns*
IFNy	1009 (3656)	1627 (4637)	775 (2569)	2577 (2322)	31.6 (956)	<0.001	ns*
sCD40L	7.5 (25.0)	35.6 (59.0)	26.6 (27.3)	19.5 (53.5)	0.0 (43.2)	0.003	0.005
TNFa	192 (407)	346 (713)	213 (990)	395 (787)	7.6 (166)	<0.001	ns*
IL-2	5.9 (9.3)	7.1 (8.1)	15.7 (15.1)	6.1 (8.9)	4.7 (4.6)	0.107	ns*
IL-9	11.7 (39.7)	23.5 (155)	20.3 (22.9)	23.6 (115)	2.8 (14.5)	0.018	ns*
IL-13	230 (203)	262 (212)	248 (371)	441 (272)	72.8 (100)	0.002	ns*
MIPa	51.1 (657)	112 (645)	252 (427)	692 (540)	15.5 (399)	0.034	ns*
MIPb	1691 (1856)	2306 (1939)	1754 (1618)	2862 (4195)	258 (1765)	0.034	ns*
TGFb1	-959 (2759)	-2083 (2243)	-1914 (1869)	-2749 (3429)	-3680 (1438)	0.033	ns*
TGFb2	-43.2 (61.7)	5.7 (46.4)	7.5 (111)	21.0 (45.0)	-34.7 (61.1)	0.132	ns*
TGFb3	4.7 (23.0)	-10.5 (19.3)	-7.2 (8.3)	-20.5 (15.3)	-10.3 (43.0)	0.869	ns*

*KruskalWallis test across all exposed groups was not significant (below *p* = 0.05)

Values are pg/mL, median (SD) in stimulated cultures after subtracting measurements in unstimulated cultures. Values are given for analyte measurements that were of sufficient quality for further analysis

**Fig. 2:**
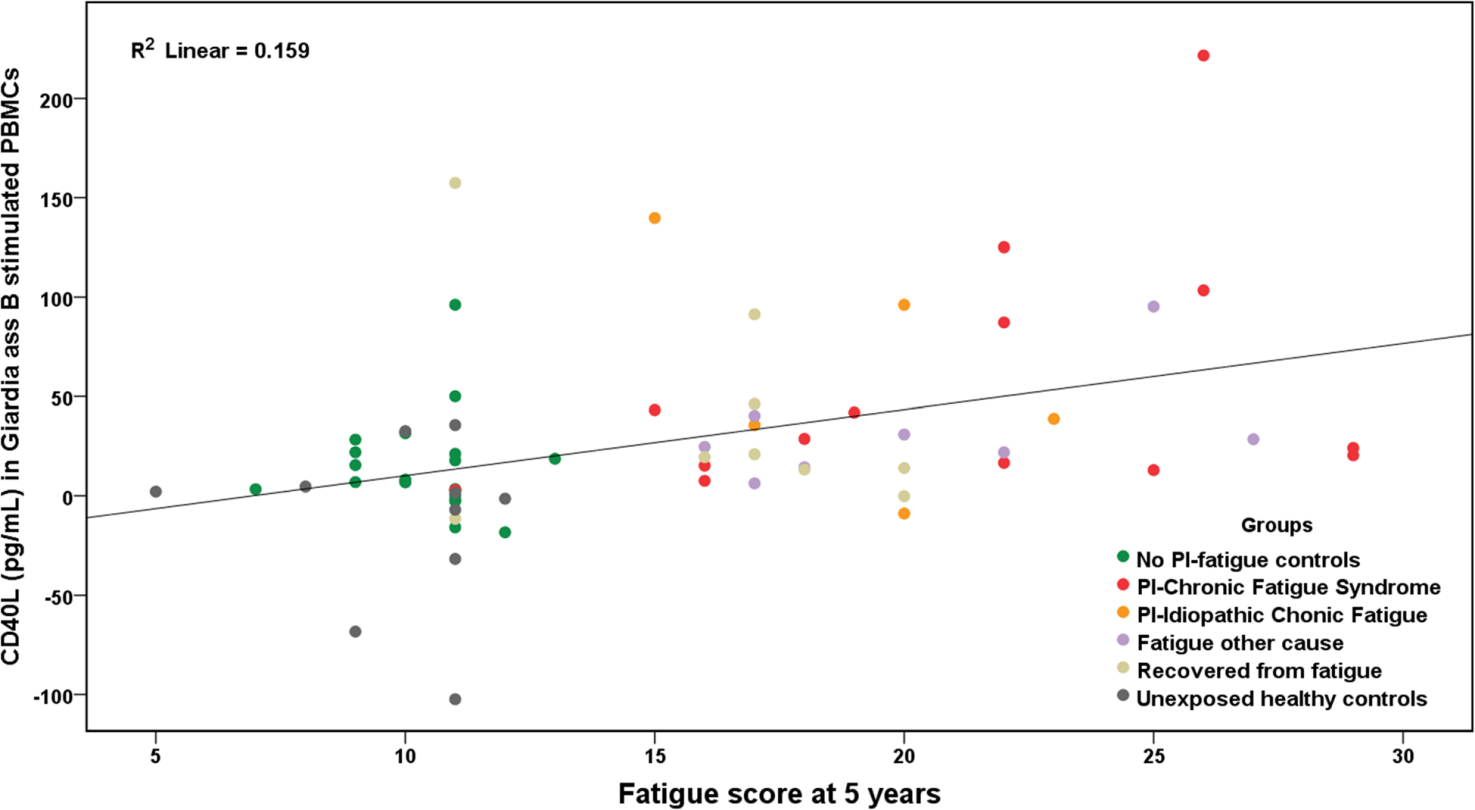
Levels of the costimulatory molecule sCD40L (CD154) found in supernatants of PBMC cultures after six days stimulation with *Giardia* assemblage B lysate correlates with fatigue scores of participants by linear regression analysis (*p* = 0.001)

In the analysis of *Giardia* antigen induced differences in cytokine profiles with regard to fatigue outcome we found only sCD40L to be significantly different within exposed groups (KruskalWallis *p* = 0.03) and the PI-CFS/PI-ICF groups having significantly elevated levels of sCD40L compared to the no PI-fatigue groups (Table 4). Also a significant difference was found when comparing the no-PI-fatigue group with the PI-CFS group (*n* = 14) alone, and with the fatigue other cause group (*p* = 0.038), but not with the recovered group. Interestingly, sCD40L levels in supernatants correlated well with fatigue scores (*p* = 0.001), shown in Fig. 2.

The antigen-specific immune responses were also explored with regard to co-morbidity with functional gastrointestinal disorders, but significant correlations were not found (data not shown).

## Discussion

The data presented in this study confirm previous finding of long term T cell memory responses towards *Giardia* [17], and bring new data on cytokines profiles elicited in this response. However, in the present study, performed in a well-defined group of patients with clinically observed post-infectious FGID and CFS five years after a common eliciting infection, we did not identify any differences in the antigen-specific cellular immunity against the culprit infectious agent, in this case *G. lamblia*. The higher levels of sCD40L in supernatants in *Giardia* stimulated PBMCs from persons with PI-CFS correlated well with fatigue scores, but could be unspecific for PI-CFS as it was also found in persons reporting fatigue that could be explained by other conditions. An alternative explanation could be that also these could have a component of *Giardia* induced fatigue, but they did not fulfil the Fukuda criteria due to their other illness. As such, further investigation is warranted to evaluate whether sCD40L might be a marker of fatigue in general or a marker for PI-CFS. Alternatively, this may be an effect related to *Giardia* exposure in all the fatigued patients. Since we did not include a control group of fatigued patients without *Giardia* exposure, we cannot conclude firmly on these options.

The present study thus underlines the need to include patient control groups where fatigue is a common symptom in all studies looking for biomarkers of CFS. Our study design, which included exposed groups with fatigue, but not fulfilling the criteria for CFS/ICF allowed for a more cautious interpretation. A previous study has found that an increase in serum sCD40L eight hours after exertion correlated with increases in physical fatigue 48 h post-exertion [18]. Our data support that CD40L, a co-stimulatory molecule which is found on a variety of cells and promotes B cell maturation, could be a marker of fatigue. Another study has suggested that sCD40L can increase blood brain permeability in vivo, and this could be a mechanism deserving further research [19].

Few studies are available to compare our data with. In line with the present study an analysis of specific humoral immunity in PI-CFS after parvovirus B19 infection did not identify any pattern distinguishing this illness [20]. Epstein-Barr virus specific cellular immune responses have been investigated in a CFS patient group of uncharacterized etiology, finding lower levels of polyfunctional ebna-1 specific T cells, whereas no differences in cytomegalovirus-responses were detected [21].

Curriu et al. found general cellular proliferative responses to be decreased in PBMCs from CFS patients [22], possibly due to increased number of regulatory T cells. We did observed a weaker proliferative response to strong T cell activation with anti-CD3/anti-CD28 stimulation in the CFS/ICF group, but these results were not statistically significant. Additionally, we also observed weaker, but non-significant, proliferative responses in the other exposed groups.

Our patient cohort offered a rare chance to study cellular immune responses to the eliciting pathogen. However, participants were recruited from a group of people who had a common exposure five years previously, and were therefore limited in number. We included those who were willing to participate, and had no chance to expand recruitment. From talking to individuals who did not wish to participate we learned that many of these experienced a substantial improvement in their condition between three and five years after the outbreak.

There was a large variation observed in measurements, causing large SDs. Some of this is likely due to the multiplicative effect of even small differences after culturing cells for six days.

The cytokine IL-17 which we have shown to play a role in human memory responses against *Giardia* infections [23] had high background levels in supernatants in the present study, leaving too few values measurable, and could not be analyzed.

Examination at an earlier time point where we would have been able to recruit more individuals still fulfilling the CFS criteria into the study could have increased the statistical power, and could possibly have resulted in stronger, more distinct immune responses.

## Conclusion

While these data confirm previous finding of T cell memory responses towards *Giardia* and bring new data on cytokine profiles, they did not reveal a difference in the magnitude of *Giardia*-specific T cell responses with respect to post-giardiasis CFS. Neither were differences in the quality of these responses, as measured by cytokine profiles in supernatants above *Giardia* stimulated PBMCs, found, except for sCD40L being elevated in the PI-CFS/ICF group as well as other exposed cases who experienced fatigue after the outbreak *Giardia* assemblage B infection.

## Additional file

Additional file 1: Table S1. Cultured PBMC supernatant analysis. Analyses of cytokines and soluble CD40L in supernatants of PBMC cultured for 6 days stimulated with *Giardia lysates*. (*PDF 72 kb*)

## Abbreviations

ATCC: American type culture collection
CFS: Chronic fatigue syndrome
EBV: Epstein barr infection
FGID: Functional gastrointestinal disorder
HIV: Human immunodeficiency virus
ICF: Idiopathic chronic fatigue
IL: Interleukin
LPS: Lipopolysaccaride
PBMC: Peripheral blood mononuclear cells
PBS: Phosphate buffered saline
PCR: Polymerase chain reaction
PI: Post-infectious
PPD: Purified protein derivate
sCD40L: Soluble cluster of differentiation 40 ligand
