# Supplementary material for: Giardia-specific cellular immune responses in post-giardiasis chronic fatigue syndrome
Source: BMC Immunol. 2017 Jan 28;18:5. doi: 10.1186/s12865-017-0190-3 (PMC5279576; doi:10.1186/s12865-017-0190-3)
Supplement: Additional file 1: Table S1. — Cultured PBMC supernatant analysis. Analyses of cytokines and soluble CD40L in supernatants of PBMC cultured for 6 days stimulated with Giardia lysates. (PDF 72 kb) [file 12865_2017_190_MOESM1_ESM.pdf]

Supplementary table 1. Cultured PBMC supernatant analysis.
Analyses of cytokines and soluble CD40L in supernatants of PBMC cultured for 6 days after stimulation with *Giardia* assemblage A and B lysate in persons exposed to this pathogen in the Bergen 2004 outbreak with or without fatigue sequels, and in unexposed controls. Values are pg/mL, median (SD) in stimulated cultures after subtracting measurements in unstimulated cultures. Negative values express higher levels in unstimulated cultures. Complete list of analysed analytes, including also values for *Giardia* assemblage B given in the manuscript.

	Exposed				Unexposed	p-values	
Analyte per stimulation agent	No PI-fatigue controls (n = 19)	PI-CFS & ICF (n = 19)	Fatigue other cause (n=9)	Recovered from fatigue (n=10)	Healthy controls (n=10)	Exposed (n=56) vs unexposed (n=10)	No PI-fatigue controls (n=19) vs PI-CFS/ICF (n=19)
Giardia ass A	n=20	n=19	n=8	n=9	n=10		
IL-1b	8.3 (68.4)	3.3 (28.9)	5.9 (19.3)	13.3 (266)	1.7 (115)	0.031	ns*
IL-4	2.1 (6.1)	0.0 (4.8)	0.0 (7.9)	0.8 (5.4)	2.7 (30.1)	0.420	ns*
IL-6	535 (2539)	233 (629)	289 (685)	752 (12866)	129 (5444)	0.058	ns*
IL-10	21.3 (15.9)	18.9 (18.8)	27.7 (10.3)	26.4 (41.4)	13.4 (49.2)	0.131	ns*
IFNy	913 (5221)	601 (4973)	1364 (2354)	3401 (2971)	153 (967)	0.020	ns*
sCD40L	31.5 (34.4)	43.3 (51.0)	46.8 (20.8)	46.0 (26.6)	11.6 (49.5)	0.005	ns*
TNFa	281 (493)	174 (373)	520 (422)	525 (1019)	110 (212)	0.017	ns*
IL-2	0.0 (14.5)	0.0 (5.8)	1.4 (3.7)	-0.6 (5.1)	2.0 (6.8)	0.388	ns*
IL-9	1.6 (25.2)	3.0 (36.0)	5.1 (10.3)	9.4 (26.7)	0.4 (145)	0.442	ns*
IL-13	244 (236)	135 (181)	270 (275)	233 (156)	84 (223)	0.061	ns*
GMCSF	9.4 (142)	0.0 (74.2)	12.4 (49.7)	30.1 (153)	0.0 (78.8)	0.177	ns*
MIPa	122 (622)	56.5 (619)	196 (412)	420 (478)	31.1 (526)	0.217	ns*
MIPb	1796 (1359)	1465 (1968)	1592 (658)	2505 (1305)	442 (1983)	0.069	ns*
TGFb1	9.2 (2440)	-98.2 (2726)	-1817 (1481)	-1743 (3510)	-117 (1412)	0.468	ns*
TGFb2	6.8 (66.0)	21.6 (50.3)	-77.4 (78.2)	0.9 (72.6)	-34.6 (68.2)	0.422	ns*
TGFb3	9.4 (20.5)	-0.7 (19.3)	-8.2 (17.8)	-20.4 (31.1)	14.3 (299)	0.076	ns*
Giardia ass B	n=20	n=19	n=8	n=9	n=10		
IL-1b	8.4 (63.5)	23.7 (211)	10.0 (37.0)	37.3 (207)	0.8 (8.8)	<0.001	ns*
IL-4	4.1 (6.6)	4.9 (4.8)	-0.7 (7.4)	7.7 (5.6)	4.0 (29.4)	0.838	ns*
IL-6	496 (8364)	781 (18566)	957 (659)	741 (991)	85.3 (310)	<0.001	ns*
IL-10	19.7 (23.3)	25.2 (354)	22.7 (13.2)	25.1 (5.9)	7.1 (25.3)	<0.001	ns*
IFNy	1009 (3656)	1627 (4637)	775 (2569)	2577 (2322)	31.6 (956)	<0.001	ns*
sCD40L	7.5 (25.0)	35.6 (59.0)	26.6 (27.3)	19.5 (53.5)	0.0 (43.2)	0.003	0.005
TNFa	192 (407)	346 (713)	213 (990)	395 (787)	7.6 (166)	<0.001	ns*
IL-2	5.9 (9.3)	7.1 (8.1)	15.7 (15.1)	6.1 (8.9)	4.7 (4.6)	0.107	ns*
IL-9	11.7 (39.7)	23.5 (155)	20.3 (22.9)	23.6 (115)	2.8 (14.5)	0.018	ns*
IL-13	230 (203)	262 (212)	248 (371)	441 (272)	72.8 (100)	0.002	ns*
MIPa	51.1 (657)	112 (645)	252 (427)	692 (540)	15.5 (399)	0.034	ns*
MIPb	1691 (1856)	2306 (1939)	1754 (1618)	2862 (4195)	258 (1765)	0.034	ns*
TGFb1	-959 (2759)	-2083 (2243)	-1914 (1869)	-2749 (3429)	-3680 (1438)	0.033	ns*
TGFb2	-43.2 (61.7)	5.7 (46.4)	7.5 (111)	21.0 (45.0)	-34.7 (61.1)	0.132	ns*
TGFb	4.7 (23.0)	-10.5 (19.3)	-7.2 (8.3)	-20.5 (15.3)	-10.3 (43.0)	0.869	ns*
Tuberculin (PPD)	n=16	n=14	n=8	n=8	n=9		
IL-1b	171 (494)	123 (422)	152 (322)	235 (328)	64.6 (142)	0.144	ns*
IL-4	0.6 (7.9)	0.0 (7.1)	0.1 (3.7)	1.2 (4.2)	1.9 (27.6)	0.674	ns*
IL-6	46299 (15478)	43172 (21101)	47856 (16730)	43910 (16441)	38190 (19503)	0.073	ns*
IL-10	141 (230)	140 (180)	134 (66)	112 (228)	61 (156)	0.251	ns*
IFNy	5696 (7773)	3699 (6334)	8921 (5771)	9696 (4271)	751 (3774)	0.040	ns*
sCD40L	51.3 (41.9)	68.1 (45.9)	110 (108)	86.1 (21.8)	36.1 (35.4)	0.027	ns*
TNFa	841 (2117)	983 (1096)	923 (928)	1257 (1527)	417 (596)	0.106	ns*
IL-2	0.0 (5.3)	0.0 (7.1)	0.5 (6.2)	-3.8 (8.5)	0.0 (10.3)	0.258	ns*
IL-9	41.9 (84.2)	125 (212)	94.0 (192)	68.5 (407)	42.7 (307)	0.587	ns*
IL-13	183 (200)	287 (427)	197 (169)	165 (90.6)	157 (647)	0.837	ns*
GMCSF	107 (156)	251 (670)	185 (165)	164 (134)	60 (2032)	0.783	ns*
MIPb	3872 (2450)	3769 (1893)	4863 (2265)	3652 (29230)	4092 (1984)	0.820	ns*
S.typhi lipopolysaccharide	n=14	n=12	n=7	n=8	n=10		
IL-1b	1465 (1142)	1330 (1220)	1172 (964)	1101 (910)	1176 (2385)	0.650	ns*
IL-4	9.3 (96.0)	10.1 (20.2)	0.0 (10.8)	0.0 (36.2)	0.0 (26.0)	0.443	ns*
IL-6	255061 (95719)	224751 (106951)	248922 (28293)	242734 (48587)	217929 (95593)	0.226	ns*
IL-10	537 (521)	457 (628)	358 (315)	471 (847)	715 (1598)	0.385	ns*
IL-17A	2085 (6274)	547 (4335)	2854 (2242)	3358 (4706)	990 (1637)	0.199	ns*
IL-22	441 (1105)	0.0 (827)	0.0 (224)	773 (633)	0.0 (543)	0.287	ns*
IFNy	38092 (33960)	10727 (19170)	24174 (16386)	37961 (28876)	6341 (18285)	0.288	ns*
sCD40L	525 (557)	231 (417)	141 (351)	487 (279)	305 (566)	0.932	ns*
TNFa	1852 (1161)	3143 (2427)	1745 (894)	2537 (3840)	1864 (5324)	0.618	ns*
IL-2	4.7 (1427)	0.0 (84.7)	11.0 (11.4)	27.6 (39.3)	0.0 (69.1)	0.249	ns*
IL-9	126 (781)	25.1 (718)	31.7 (227)	321 (465)	44.1 (157)	0.403	ns*
IL-13	235 (941)	113 (721)	73.0 (275)	346 (650)	132 (316)	0.309	ns*
GMCSF	343 (919)	250 (988)	296 (140)	455 (422)	193 (301)	0.314	ns*
MIPb	22750 (24459)	4626 (24025)	41757 (26924)	18875 (34739)	10172 (20709)	0.367	ns*

* KruskalWallis test across all exposed groups was not significant (below p=0.05). ns= not significant
